# New Morphiceptin Peptidomimetic Incorporating (1*S*,2*R*,3*S*,4*S*,5*R*)-2-Amino-3,4,5-trihydroxycyclopen-tane-1-carboxylic acid: Synthesis and Structural Study

**DOI:** 10.3390/molecules25112574

**Published:** 2020-06-01

**Authors:** Raquel Soengas, Marcos Lorca, Begoña Pampín, Víctor M. Sánchez-Pedregal, Ramón J. Estévez, Juan C. Estévez

**Affiliations:** 1 Departmento de Química Orgánica e Inorgánica, Universidad de Oviedo, Julián Clavería 8, 33006 Oviedo, Spain; rsoengas@uniovi.es; 2 Centro Singular de Investigación en Química Biolóxica e Materiais Moleculares (CIQUS), Universidade de Santiago de Compostela, 15782 Santiago de Compostela, Spain; marcos.lorca@gmail.com; 3 Departamento de Química Orgánica, Universidade de Santiago de Compostela, 15782 Santiago de Compostela, Spain; begona.pampin@galchimia.com (B.P.); victor.pedregal@usc.es (V.M.S.-P.)

**Keywords:** morphiceptin, alicyclic β-amino acids, peptidomimetics, nitro sugars, analgesic

## Abstract

We present the synthesis and structural study of a new peptidomimetic of morphiceptin, which can formally be considered as the result of the replacement of the central proline residue of this natural analgesic drug with a subunit of (1*S*,2*R*,3*S*,4*S*,5*R*)-2-amino-3,4,5-trihydroxycyclopentane-1-carboxylic acid, previously obtained from *L*-idose. An optimized synthesis of this trihydroxylated cispentacin derivative is also reported. Molecular docking calculations on the target receptor support a favorable role of the hydroxy substituents of the non-natural β-amino acid incorporated into the peptidomimetic.

## 1. Introduction

**Figure 1:**
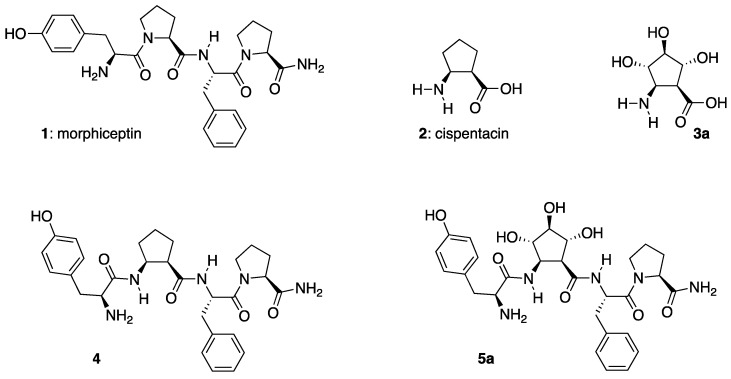
Cispentacin, (*1S,2R,3S,4S,5R*)-3,4,5-trihydroxycispentacin (**3a**) and their respective morphiceptin peptidomimetics **4** and **5a**.

Research on proteins as therapeutics is a topic of great current interest [1]. After insulin therapy was authorized in the 1920s, more than 60 peptides have been approved as potent bioactive agents, and much more have entered clinical studies. Specifically, morphiceptin (**1**), a tetrapeptidic amide (Tyr-Pro-Phe-Pro-NH_2_) [2] structurally related to β-casomorphin and originally isolated from bovine β-casein [3], was shown to be a highly selective opioid peptide agonist for μ-receptor [4]. However, the use of α-peptides as drugs suffers from limitations derived from their reduced diversity both in terms of primary and secondary structures, the presence of a side effects due to their conformational flexibility, the short half-life and the oral bioavailability [5]. Approaches conceived for overcoming these limitations include structural modification strategies [6,7]. β-Amino acids have proven to be suitable candidates for this purpose. In fact, the replacement of specific α-amino acids in α-peptides by β-amino acids usually result in α/β-peptides more resistant to protease and peptidase degradation [8,9,10]. Moreover, the increased conformational stability of these peptidomimetics facilitates their interaction with receptors and enzymes [11,12]. Cyclopentane β-amino acids are ideal candidates for conformational stabilization of peptides [13,14], a fact that has been related to the strong rigidity of their homooligomers [15]. Special interest received by (1*R*,2*S*)-2-aminocyclopentanecarboxylic acid (cispentacin **2**, Figure 1), a natural compound isolated from the cell broth of *Bacillus cereus* or *Strepomyces setonii*, is due to its potent antifungal activity in vivo against *Candida albicans* [16,17].

Opioid receptors are extremely important targets in medicinal chemistry because of their essential role in the perception of pain. Among the three major subtypes (μ, κ and δ) of this family of receptors, the μ group represents the major target of the analgesics. Although several endogenous peptide ligands of opioid receptors are known, morphiceptin **1** (Tyr-Pro-Phe-Pro-NH_2_) (Figure 1) is one of the few that show significant μ-selective agonistic activity [18]. Nevertheless, its easy metabolic degradation promoted studies on morphiceptine peptidomimetics [19,20,21], including tetrapeptide **4** (Figure 1) resulting from the substitution of the central proline subunit of morphiceptin by cispentacin, which was ∼six times more active at the μ-receptor and ∼20-times more active at the δ-receptor than the parent morphiceptin [22,23].

In view of the interest of peptides based on cyclopentane β-amino acids, a few years ago our research group started a program aimed at increasing the limited number of this class of β-amino acids. This led us to develop a stereospecific synthesis of polyhydroxylated cyclopentane β-amino acids [24,25,26,27,28,29], including the polyhydroxylated cispentacin (Pcp) **3a** (Figure 1) [26]. Despite their limited presence in the literature [30], these polyhydroxylated constrained alicyclic β-amino acids open opportunities for accessing to lipo or hydrosoluble β-peptides, by the protection or deprotection of the hydroxy substituents on the cyclopentane ring.

We present herein an optimized synthesis of polyhydroxylated cispentacin **3a** from l-idose and the synthesis and structural study of the new morphiceptin peptidomimetic **5a**, where the central proline subunit of morphiceptin has been replaced by compound **3a**.

## 2. Results and Discussion

### 2.1. Synthesis of the Polyhydroxylated 2-Aminocyclopentanecarboxylic acid (**3b**)

**Scheme 1:**
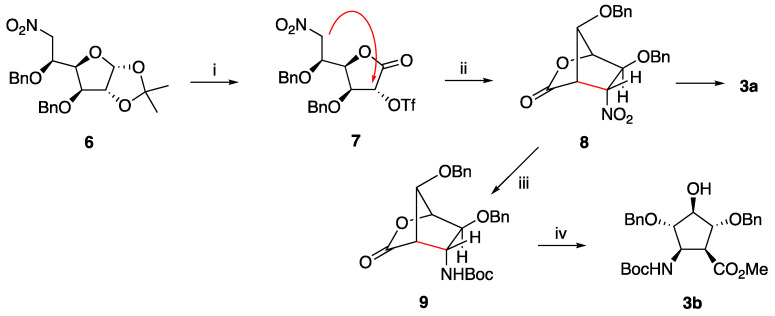
*Reagents and conditions*: (i) [25]. (ii) Tetrabuthyl Ammonium Fluoride (TBAF), Tetrahydrofuran (THF), rt, 4 h, 81%. (iii) H_2_, Ni-Raney 10% in H_2_O, Boc_2_O, EtOAc, rt, 4 h, 57%. (iv) NaOMe, MeOH, −10 °C, 30 min, 90%.

Proceeding as before [25], fluoride-promoted intramolecular *C*-alkylation of *L*-iduronolactone triflate **7**, provided the known bicyclic lactone **8** in 81% yield (previously reported yield: 53%) (Scheme 1, which was then transformed into the polyhydroxylated cispentacin (Pcp) **3a** (the first reported member of this family of amino acids having a *cis* disposition of its amino and carboxyle moieties). The present yield improvement in **8** was achieved after the careful drying of compound **7**. When the bicyclic lactone **8** was now subjected to catalytic hydrogenation with Raney-Ni, in the presence of *t*-butoxycarbonyl anhydride, and the resulting *N*-Boc protected bicyclic aminolactone **9** was reacted with sodium methoxide in methanol, the orthogonally protected Pcp ester **3b** was obtained as a result of the opening of the lactone moiety.

Our previous studies on the key intramolecular *C*-alkylation of nitronates involved in the stereoselective synthesis of polyhydroxylated cyclopentane β-amino acids established that the efficiency of this key cyclization depends on several structural factors, including the sp^2^ or sp^3^ character of the carbon atom at C-1. In general, better results were achieved with substrates bearing an sp^3^ hybridized anomeric carbon rather than with their sp^2^ counterparts.

**Scheme 2:**
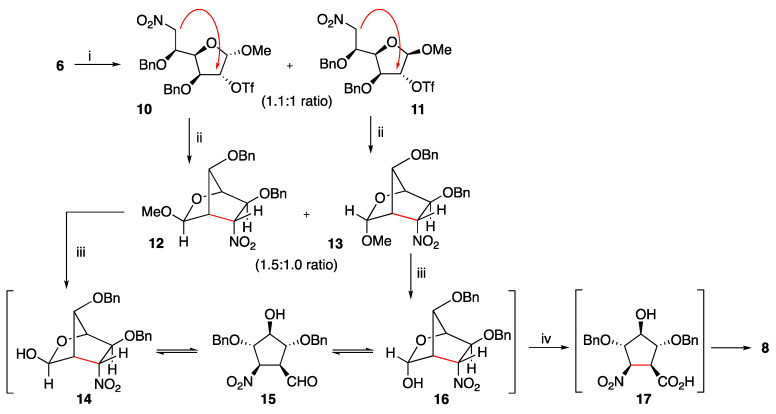
*Reagents and conditions*: (**i**) [25]. (**ii**) TBAF, THF, rt, 6 h, 87%. (**iii**) TFA/H_2_O 1:1, rt, 12 h (**iv**) Br_2_, BaCO_3_, 1,4-dioxane, rt, 4 h, 76% (two steps).

Looking for further improvement in the yield in **3b**, we also revisited the previously reported intramolecular *C*-alkylation of *L*-idofuranose triflates **10** and **11** (Scheme 2). Thus, when a solution of an under high vacuum overnight dried 1.1:1 mixture of triflates **10** and **11** in THF was treated with TBAF, a 1.5:1.0 mixture of epimeric bicyclic nitrolactol glycosides **12** and **13** was obtained in 87% yield (previously reported yield: 35%).

Furthermore, anomers **10** and **11** were independently subjected to the C-alkylation protocol. The reaction of *O*-triflyl derivative **10** with TBAF in THF gave the expected nitro bicyclic nitroglycoside **12** (90% yield). Similarly, the minor anomer **11** gave bicycle **13** (66% yield). The *exo* disposition of the anomeric methoxy substituent of compound **12** was established from the ^1^H NMR spectra, which displays a singlet at 4.57 ppm for H-3. On the other hand, the *endo* disposition of the methoxy substituent of anomer **13** was deduced from a doublet at 4.99 ppm present in its ^1^H NMR spectra (H-3, *J*_3,4_ = 2.6 Hz) and confirmed through an X-ray crystallographic study [25].

To sum up, nitrolactone **9** was obtained in 81% yield, *exo*-nitrolactol **12** in 90% yield and *endo*-nitrolactol **13** in 66% yield. These results are in accordance with our previous studies with d-glucose. The higher yield achieved for compound **12** with respect to compound **9** was related to the sp^3^ and sp^2^ character of the carbon atoms at C-1 position of their respective precursors 1**0** and **7**. The greater flexibility of the five membered ring of compound **10** facilitates the intramolecular attack by the nitronate on the carbon atom at C-2, bearing the -*O*Tf leaving group. The moderate yield achieved for compound **13** was attributed to the fact that a similar attack on **11** is perturbed by the spatial orientation of its methoxy substituent at C-1.

Treatment of the mixture **12** + **13** with aqueous trifluoroacetic acid provided a tricomponent mixture of the corresponding lactols **14** and **16** (major components), which are in equilibrium with its open form **15** (minor component). The direct oxidation of this mixture with bromine in the presence of barium carbonate provided the bicyclic nitrolactone **8** in a 74% yield. The formation of this compound probably involves the oxidation of aldehyde **15** to its carboxylic acid **17** and subsequent lactonization of this intermediate under the reaction conditions.

In summary, orthogonally protected amino acid **3b** was obtained from nitrosugar **6** via nitrolactone **8** in five steps with a 34% overall yield. Alternatively, **3b** can be prepared using nitroglucofuranosides **10** and **11**, even though the intramolecular cyclization of these glycosides is more efficient, this route involves a seven synthetic steps route and affords amino acid **3b** in a 31% global yield. Accordingly, the former strategy was more efficient and thence more suitable for our synthetic purposes.

### 2.2. Preparation of the Morphiceptin Peptidomimetic **5a**

**Scheme 3:**
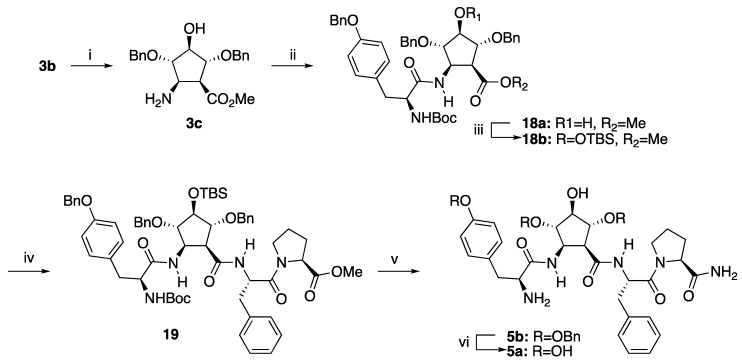
*Reagents and conditions*: (**i**) 1. TFA, CH_2_Cl_2_, rt, 15 min; (**ii**). Boc-Tyr-(Bn)-OH, TBTU, DIEA, CH_2_Cl_2_, rt, 12 h, 77% (2 steps). (**iii**) DMF, rt, 12 h, 93%. (**iv**) 1. LiOH, MeOH, H_2_O, rt, 2 h 2. Phe-Pro-OMe, TBTU, Et_3_N, DMF, 47% (2 steps). (**v**) 1. LiOH, MeOH, H_2_O, rt, 2 h 2. 30% aq. NH_3_, ClCO_2_Et, Et_3_N, THF, rt., 12 h 3. TFA/CH_2_Cl_2_ 2:3, rt, 15 min., 40% (3 steps). (**vi**) H_2_, Pd(OH)_2_, 20 atm, MeOH, 98%.

We envisioned the preparation of morphiceptine peptidomimetic **5a** from amino acid **3b** according to the synthetic route depicted in Scheme 3. The removal of the Boc group of **3b** by treatment with trifluoracetic acid and subsequent reaction of the resulting amino acid ester **3c** with BocTyr(Bn)-OH, using TBTU as a coupling promoter, provided dipeptide BocTyr(Bn)-Pcp(Bn)-OMe **18a**. The formation of the desired dipeptide is supported by the ESI-HRMS spectrum, which displays an intense parent ion at *m/z* 725.3431, corresponding to the [M + H]^+^ (calculated for C_42_H_49_N_2_O_9_ [M + H]^+^: 725.3432). As a further confirmation, the ^13^C NMR spectrum shows a signal at 157.6 ppm corresponding to the carbonyl of the carbamate protecting group and two signals at 171.1 and 172.4 ppm corresponding to the carbonyl groups of both the ester and amide moieties.

In order to prevent a potential lactonization, the free hydroxy group of dipeptide **18a** was protected by treatment with *t*-butyldimethylsilylchoride in the presence of imidazole. The basic hydrolysis of the ester group of the resulting dipeptide **18b**, followed by the coupling of the resulting carboxylic acid with dipeptide Phe-Pro-OMe in the presence of TBTU, afforded tetrapeptide BocTyr-(Bn)-Pcp(Bn, TBS)-Phe-Pro-OMe (**19)** in a 47% yield. The formation of tetrapeptide was confirmed by the ESI-HRMS spectrum, which displays an intense parent ion at *m/z* 1083.5493 corresponding to the [M + H]^+^ (calculated for C_61_H_75_N_4_O_12_Si [M + H]^+^: 1083.5509). The presence of the methyl ester and the three amide bonds was ascertained in the ^13^C NMR spectrum from the signals at δ 170.0, 170.7, 171.5 and 172.4 ppm corresponding to the carbonyl carbons.

The basic hydrolysis of the ester group of peptide **19,** followed by the reaction of the resulting carboxylic acid with ammonia in the presence of ethyl chloroformate and subsequent acidic hydrolysis resulted in the formation of tetrapeptide **5b** in a 41% yield. The formation of this tetrapeptide was deduced from the [M + H]^+^ ion present in the ESI-HRMS spectrum at *m/z* 854.4133 (calculated for C_50_H_56_N_5_O_8_ [M + H]^+^: 854.4123). The removal of the *t*-butyldimethylsilyl and benzyloxy carbonyl groups of **19** was further confirmed from the ^13^C NMR spectrum where both the signals of the SiMe_2_ at –4.5/–4.7 ppm and the carbonyl of the carbamate moiety at 157.0 ppm showed by its precursor **19** were not present.

Finally, the removal of the benzyl protecting groups under catalytic hydrogenation gave the desired morphiceptin peptidomimetic **5a** (14 steps from nitro sugar 6, 4.6% overall yield), as deduced from the ESI-HRMS spectrum, which displays a strong parent ion corresponding to [M + H]^+^ at 584.2723 (calculated for C_29_H_38_N_5_O_8_ [M + H]^+^: 584.2715).

### 2.3. Docking Studies

Molecular docking calculations provide us with a tool to simulate the atomic level interaction between a ligand and its receptor. The understanding of the binding modes with the target receptor is crucial for the development of drugs with enhanced potency and selectivity. In this study, comparative docking analysis of natural morphiceptin **1**, cispentacin peptidomimetic **4** and the newly developed polyhydroxylated cispentacin (Pcp) peptidomimetic **5a** into the μ-opioid receptor (MOR) (PDB ID: 6DDF) was done.

**Figure 2:**
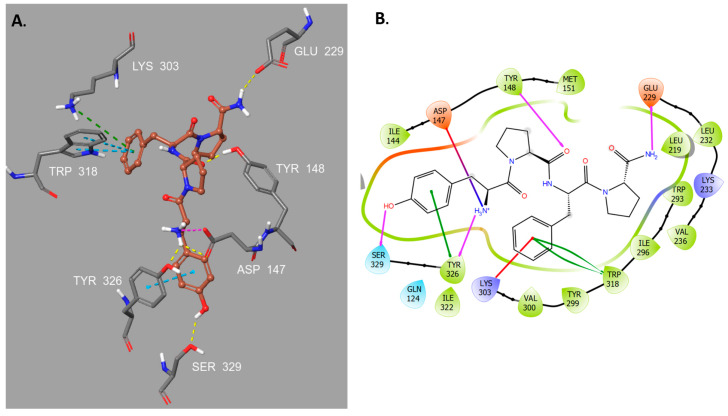
(**A**) Binding mode of morphiceptin **1**. Blue lines indicate π-cation interactions, and purple lines indicate salt bridges. The ligand is represented in brown and the receptor residues are represented in gray. (**B**) Two-dimensional diagram of the main interactions between morphiceptin and the residues involved in ligand recognition of the μ-opioid receptor (MOR) binding site. Purple lines represent hydrogen bonds, blue-red represent salt bridges, red lines represent π-cation, and green lines represent π-stacking. Hydrophobic residues are colored in green, negatively charged in red, polar in light blue and positively charged in purple.

Morphiceptin **1** is anchored to the binding pocket by a persistent salt bridge between the α-amino cationic group of the tyrosine residue and Asp147, and a hydrogen bond between this same group and Tyr326 (Figure 2A,B).

The presence of the central proline residue gives morphiceptin a certain degree of rigidity. This limited conformational freedom facilitates the binding to the MOR active site through π-stacking and π-cation interactions between the phenylalanine of morphiceptin and the residues Trp318 and Lys303. Furthermore, the carbonyl of the amide group of morphiceptin central proline is linked through a hydrogen bond to Tyr148.

**Figure 3:**
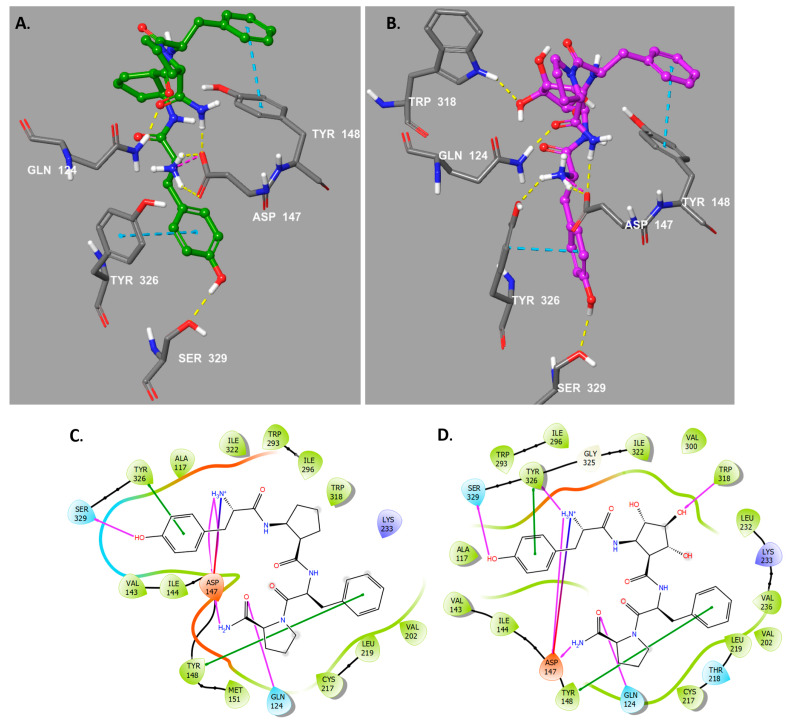
(**A**) Binding mode of compound **4**. (**B**) Binding mode of compound **5a**. Blue lines indicate π-cation interactions and purple lines salt bridges. Residues of the receptor MOR are represented in gray, **4** in green and **5a** in purple. (**C**) Two-dimensional diagram of the main interactions between **4** and the residues involved in ligand recognition of the MOR binding site. (**D**) Two-dimensional diagram of the main interactions between **5a** and the residues involved in ligand recognition of the MOR binding site. Purple lines represent hydrogen bonds, blue-red represent salt bridges, red lines represent π-cation, and green lines represent π-stacking. Hydrophobic residues are represented in green, negatively charged in red, polar in light blue and positively charged in purple.

Peptidomimetics **4** and **5a** bind to MOR in a manner similar to morphiceptin, as both retain the principal interactions for the binding of morphiceptin: the hydrogen bond between the α-amino cationic group of the tyrosine residue with Asp147, and the hydrogen bond of the tyrosine hydroxyl group with Ser329 (Figure 3).

The main difference lies on the greater conformational flexibility of peptides **4** and **5a** compared to morphiceptin. Due to this flexibility, peptide **4** adopts an extended conformation in the MOR pocket, enabling the hydrogen bond interaction of the -NH_2_ group of its terminal proline amide with the carbonyl of Asp147 (Figure 3A,C). Furthermore, due to this extended conformation, the carbonyl of the terminal proline amide participates as an acceptor of a hydrogen bonding interaction with the polarized proton of Gln124 side chain. However, the extended conformation increases the distance with Tyr326, preventing the hydrogen bond interaction with the α-amino cationic group of the tyrosine residue (Figure 3A,C). Peptidomimetic **5a** also adopts an extended conformation and, like peptide **4**, presents the hydrogen bond interaction of the amide NH_2_ group of the terminal prolin with the carbonyl of Asp147 and with the polarized proton of Gln124 side chain, as well as the π-stacking interaction between the tyrosine and Tyr326 (Figure 3B,D). Interestingly, due to the hydroxyl groups of the cyclopentane ring, compound **5a** accommodates better in the binding pocket by establishing a new hydrogen bond interaction between a hydroxy group of the central Pcp residue and the polarized HN proton of the Trp318 indole ring. This additional interaction increases the electrostatic energy of the complex and, besides, approximates the peptide to the Tyr326 residue of the binding site, so that the hydrogen bond interaction with Tyr326 that was lost in peptide **4** is now recovered in peptide **5a**.

### 2.4. Conformation of the Morphiceptin Peptidomimetic **5a** in Solution

**Figure 4:**
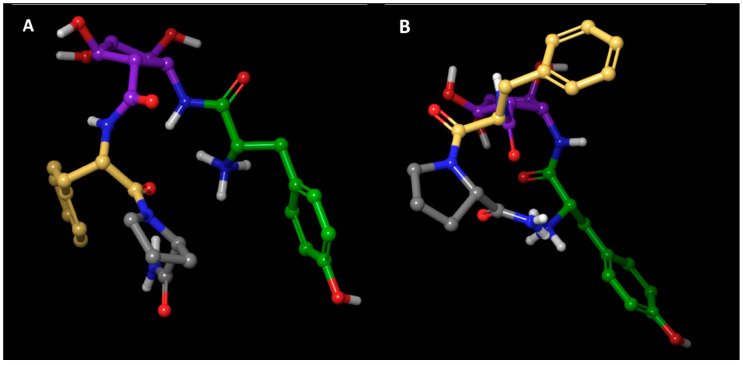
Comparison of the calculated conformations of **5a**. (**A**) Free in solution. (**B**) Bound to the receptor. H white, O red, N blue, Tyr1 green, Pcp2 purple, Phe3 yellow, Pro4 grey.

As the 3D structure of the ligand/receptor complex was not accessible experimentally, we studied the structure of peptide **5a** free in solution by NMR spectroscopy at 500 MHz in DMSO-d_6_ (Figures S5–S12, Supplementary Materials). The ^1^H NMR spectrum indicated the presence of a major conformer, together with at least two minor conformers in slow conformational equilibrium (Figure S5, Supplementary Materials). The structure in solution was derived by the analysis of the H-H distances determined from the 2D ROESY spectrum (Table S2, Supplementary Materials). Two inter-residual NOE contacts (Pcp2:HE2/Phe3:HB1 and Phe3:HA/Pro4:HD1 of medium and strong intensities, respectively) support that the backbone is partially folded, at least in some conformers of the solution ensemble. Figure 4 depicts the calculated conformation that satisfies all observed NOEs, determined by restrained molecular dynamics [31]. The solution conformation differs from the bioactive or receptor-bound conformation in the orientation of the Phe3 and Pro4 residues relative to Pcp2. Conversely, the receptor-bound conformation does not satisfy the inter-residual NOEs observed in DMSO solution. If we assume that the conformational ensemble in DMSO is representative of that in aqueous solution, the docking model supports that conformation of **5a** changes upon binding to the receptor. Given that NMR gives evidence that molecule **5a** is flexible when free in solution, the fast-exchange equilibrium of totally or partially extended conformers, or even a certain population of the bioactive conformation, cannot be ruled out.

## 3. Materials and Methods

### 3.1. Chemistry

All new compounds were characterized by NMR spectroscopy and high-resolution mass spectrometry. NMR spectra were recorded on Bruker Avance III HD 300 (Bruker, Billerica, MA, USA) (^1^H 300.13 MHz; ^13^C 75.47 MHz) and Bruker Avance III HD 500 (Bruker, Billerica, MA, USA) (^1^H 500.13 MHz; ^13^C 125.76 MHz) spectrometers and processed with MestreNova. The following abbreviations are used to indicate the multiplicity of signal: s—singlet, bs—broad singlet, d—doublet, t—triplet, q—quartet and sep—septet. High-Resolution Mass Spectra (HRMS) were recorded on a Hewlett Packard 5988A mass spectrometer (Hewlett Packard, Palo Alto, CA, USA) using electrospray ionization. Specific rotations were recorded on a JASCO DIP-370 optical polarimeter (JASCO, Inc., Easton, MD, USA). Elemental analyses were obtained from the Elemental Analysis Service at the University of Santiago de Compostela. Thin layer chromatography (TLC) was performed using Merck GF-254 type 60 (Merck KGaA, Darmstad, Germany) silica gel and ethyl acetate/hexane mixtures as eluants; the TLC spots were visualized with Hanessian mixture. Column chromatography was carried out using Merck type 9385 (Merck KGaA, Darmstad, Germany) silica gel.

#### 3.1.1. (*1S,4S,5R,6S,7R*)-6,7-Dibenzyloxy-5-nitro-2-oxabicyclo-[2.2.1]heptan-3-one (**8**)

Under inert atmosphere, 1 M solution of tetrabutylammonium fluoride in THF (0.45 mL) was added to a solution of the triflate **7** (dried *in vacuo* overnight) in THF (4 mL) and the mixture was stirred at rt for 4 h. The solvent was removed under reduced pressure and the residue was dissolved in dichloromethane (25 mL). The resulting solution was washed with water (3 × 13 mL) and the combined organic layers were dried with anhydrous sodium sulfate, filtered and concentrated in vacuo. The residue was purified by flash column chromatography (ethyl acetate/hexane 1:5) to give bicyclic nitrolactone **8** (0.14 g, 81%) as a yellow oil. ^1^H NMR (500 MHz, CDCl_3_): δ 3.42–3.44 (m, 1H, H7), 3.42–3.44 (m, 1H, H7), 4.37 (m, 1H, H1), 4.48–4.52, 4.65–4.79 (2 × m, 6H, 2 × –OCH_2_Ph, H5, H6), 5.45 (dd, 1H, *J*_4,7_ = 3.4 Hz, *J*_4,5_ = 3.4 Hz, H4), 7.26–7.37 (m, 10H, 10 × HAr); MS (ESI+, *m/z*, %): 370 [(M + H)^+^, 6%], 369 (M^+^, 15%), 368 [(M − H)^+^, 8%], 278 (52%), 181 (66%), 91 (100%).

#### 3.1.2. (*1S,4S,5R,6S,7R*)-6,7-Dibenzyloxy-5-t-butoxycarbonylamino-2-oxabicyclo-[2.2.1]heptan-3-one (**9**)

Ni-Raney (3.2 mL, 10% in H_2_O) and *t*-butoxycarbonyl anhydride (0.14 g, 0.62 mmol) were sequentially added to a deoxygenated solution of **8** (0.21 g, 0.57 mmol) in ethyl acetate (3 mL). The mixture was stirred at rt for 15 h under hydrogen atmosphere (1 atm). The reaction mixture was dried (Na_2_SO_4_), filtered over a pad of celite and the filtrate was concentrated to dryness under reduced pressure. The residue was subjected to flash column chromatography (AcOEt/hexane 1:3) to give amine **9** (0.14 g, 57%) as an amorphous white solid. [α]${}_{D}^{24}$: +13.3° (c 0.6, CHCl_3_). ^1^H NMR (CDCl_3_, 300 MHz, ppm): 1.46 (s, 9H, 3 × CH_3_), 3.10 (bs, 1H, H4), 4.24 (bs, 1H, H7), 4.24 (bs, 1H, H6), 4.49–4.81 (m, 7H, H1, H5, 2 × CH_2_Ph, NH), 7.29–7.31 (m, 10H, 10 × HAr). ^13^C NMR (CDCl_3_, 62.5 MHz, ppm): 28.2, 50.0, 54.0, 71.6, 71.9, 79.3, 80.2, 81.3, 83.4, 127.8, 127.9, 128.0, 128.1, 128.4, 136.5, 137.1, 154.7, 171.7. HRMS (ESI+) calculated for C_25_H_30_N_2_O_6_ [M + H]^+^: 440.2068. Found: 440.2071.

#### 3.1.3. Methyl (*1S,2R,3S,4S,5R*)-2,4-Dibenzyloxy-5-t-butoxycarbonylamino-3-hydroxycyclo-pentanecarboxylate (Pcp) (**3b**)

To a solution of bicyclic amine **9** (33 mg, 0.075 mmol) in dry methanol (1 mL) cooled at 0 °C, NaMeO was added (20 mg, 0.037 mmol) and the resulting mixture was stirred at rt for 30 min. After the evaporation of the solvent under reduced pressure, the residue was dissolved in EtOAc (10 mL), washed with water (3 × 5 mL), dried (Na_2_SO_4_), filtered and evaporated. The resulting residue was purified by flash column chromatography (EtOAc/Hex 1:3) to afford hydroxycyclopentanecarboxylate **3b** (32 mg, 0.064 mmol, 90%) as an amorphous white solid. [α]${}_{D}^{23}$: −24.9° (c 0.6, CHCl_3_). ^1^H NMR (CDCl_3_, 300 MHz, ppm): 1.38 (s, 9H, 3 × CH_3_), 2.48 (bs, 1H, OH), 3.25 (bs, 1H, H-1), 3.62 (s, 3H, OCH_3_), 3.64–3.70 (m, 1H, H4), 4.02–4.04 (m, 2H, H2, H3), 4.28–4.35 (m, 1H, H5), 4.50 (d, 1H, *J* = 12.4 Hz, CHPh), 4.55 (d, 1H, *J* = 12.4 Hz, CHPh), 4.60 (d, 1H, *J* = 11.9 Hz, CHPh), 4.68 (d, 1H, *J* = 11.9 Hz, CHPh), 4.93 (d, 1H, *J*_NH,5_ = 8.3 Hz, NH), 7.21–7.30 (m, 10H, 10 × HAr). ^13^C NMR (CDCl_3_, 62.5 MHz, ppm): 28.2, 50.7, 52.1, 53.6, 72.1, 80.0, 84.0, 86.1, 127.7, 128.4, 137.7, 137.9, 154.9, 172.8. MS (ESI+, *m/z*, %): 473 [(M + 2H)^+^, 22]; 472 [(M + H)^+^, 41]; 372 [(M − Boc)^+^, 100]; 91 [(PhCH_2_)^+^, 97]. Elemental analysis: calculated for C_26_H_33_NO_7_: C 66.22, H 7.05, N 2.97; found C 66.19, H 7.17, N 2.93.

#### 3.1.4. (*1S3S,4S,5R,6S,7R*)-6,7-Dibenzyloxy-3-methoxy-5-nitro-2-oxabicycle[2.2.1]heptane (**12**) and (*1S,3R,4S,5R,6S,7R*)-6,7-Dibenzyloxy-3-methoxy-5-nitro-2-oxabicycle[2.2.1]heptane (**13**)

Under argon atmosphere, a 1 M solution of tetrabutylammonium fluoride in THF (1.20 mL) was added to a solution of an under high vacuum overnight dried mixture of triflates **10** and **11** in THF (11 mL) and the mixture was stirred at rt for 4 h. The solvent was removed under reduced pressure and the residue was dissolved in dichloromethane (40 mL). The resulting solution was washed with water (3 × 25 mL) and the organic layers were dried with anhydrous sodium sulfate, filtered and concentrated in vacuo. The residue was purified by flash column chromatography (eluant: 1:5 ethyl acetate/hexane) to give bicyclic nitroglycosides **12** (0.220 g) and **13** (0.150 g) in a 87% combined yield. Data for **12**: ^1^H NMR (500 MHz, CDCl_3_): δ 3.10–3.13 (m, 1H, H4), 3.32 (s, 3H, OCH_3_), 4.30–4.31 (m, 1H, H1), 4.36–4.39 (m, 1H, H6), 4.46 (d, 1H, J = 10.9 Hz, CHPh), 4.57 (s, 2H, H3, H7), 4.59 (d, 1H, J = 10.9 Hz, CHPh), 4.49 (d, 1H, J = 11.8 Hz, CHPh), 4.57 (d, 1H, J = 11.8 Hz, CHPh),5.05–5.10 (m, 1H, H5), 7.24–7.31 (m, 10H, 10 × HAr). Data for **13**: [α]${}_{D}^{27}$ −45.6° (c 1.5, CHCl_3_). ^1^H NMR (500 MHz, CDCl_3_): δ 3.27 (s, 3H, OCH_3_), 3.38–3.43 (m, 1H, H4), 4.13–4.16 (m,1H, H1), 4.32 (bs, 1H, H7), 4.45 (d, 1H, *J* = 11.2 Hz, CHPh), 4.59 (d, 1H, *J* = 11.2 Hz, CHPh), 4.66–4.70 (m, 1H, H6), 4.68 (d, 1H, *J* = 11.9 Hz, CHPh), 4.76 (d, 1H, *J* = 11.9 Hz, CHPh), 4.99 (d, 1H, *J* = 2.6 Hz, H3), 5.16–5.19 (m, 1H, H5), 7.26–7.36 (m, 10H, 10 × HAr). ^13^C NMR (500 MHz, CDCl_3_): δ 47.3, 55.7, 71.8, 72.3, 77.4, 79.7, 81.3, 88.1, 101.8, 127.5, 127.6, 127.8, 127.9, 128.2, 128.2, 136.6, 137.5. MS (ESI^+^, *m/z*, %): 386 [(M + H)^+^, 14]; 385 [M^+^, 3]; 384 [(M − H)^+^, 9]; 181 (14), 91 [(CH_2_Ph)^+^, 100]. Elemental analysis: calculated for C_21_H_23_NO_6_: C 65.44, H 6.02, N 3.63; found C 65.44, H 6.34, N 3.62.

#### 3.1.5. Methyl (*1S,2R,3S,4S,5R*)-5-(*N*-tert-butoxycarbonyl-*O*-benzyloxycarbonyl-l-tyrosylamino)-2,4-dibenzyloxy-3-hydroxy-cyclopentanoate (**18a**)

To a solution of Boc-protected amine **3b** (0.12 mmol) in CH_2_Cl_2_ (1 mL), TFA was added (0.38 mL) and the mixture was stirred at rt for 30 min. After the evaporation of the solvents and coevaporation with toluene, a solution of the resulting amine **3c** and DIEA (0.075 mL, 0.42 mmol) in CH_2_Cl_2_ (1 mL) was added to a solution of BocTyr-(Bn)-OH (50 mg, 0.12 mmol) and TBTU (54 mg, 0.17 mmol) in CH_2_Cl_2_ (1 mL), previously stirred for 1 h. The reaction mixture was stirred for 6 h, when the solvents were removed under reduced pressure. The resulting residue was taken up in ethyl acetate (10 mL), washed with aqueous saturated solution of NH_4_Cl (5 mL) and brine (5 mL), dried, filtered and evaporated under reduced pressure. The residue was precipitated with ethyl acetate/hexane to obtain dipeptide **18a** (60 mg, 77%) as a white amorphous solid. ^1^H-NMR (CD_3_OD, 250 MHz, ppm): δ 1.38 (s, 9H, -C(CH_3_)_3_), 2.94–2.97 (m, 2H, Tyr-CH_2_Ar), 3.21–3.26 (m, 1H, Pcp-H2); 3.58 (s, 3H, OCH_3_), 3.71–3.75 (m, 1H, Pcp-H3), 4.03–4.07 (m, 2H, Pcp-H4, Pcp-H6), 4.15–4.24 (m, 1H, Pcp-H3), 4.56–4.70 (m, 5H, Tyr-H2, 2 × OCH_2_Ph), 4.86 (d, 1H, *J* = 7.9 Hz, Pcp-NH), 6.58 (d, 1H, *J* = 7.6 Hz, Tyr-NH), 4.96 (s, 2H, OCH_2_Ph), 6.84 (d, 2H, *J* = 8.5 Hz, 2 × HAr), 7.07 (d, 2H, *J* = 8.5 Hz, 2 × HAr), 7.25–7.38 (m, 15H, 15 × HAr). ^13^C-NMR (CD_3_OD, 62.5 MHz, ppm): δ 28.1, 37.1, 50.0, 52.2, 55.8, 69.8, 71.8, 72.0, 80.2, 80.3, 84.9, 86.2, 114.8, 127.3, 127.6, 127.7, 127.9, 128.3, 128.4, 128.5, 130.3, 136.8, 137.6, 137.9, 155.3, 157.6, 171.1, 172.4. HRMS (ESI+) calculated for C_42_H_49_N_2_O_9_ [M + H]^+^: 725.3432. Found: 725.3431.

#### 3.1.6. Methyl (*1S,2R,3S,4S,5R*)-5-(*N*-tert-butoxycarbonyl-*O*-benzyloxycarbonyl-l-tyrosylamino)-2,4-dibenzyloxy-3-tert-butyldimethylsilyloxy-cyclopentanoate (**18b**)

To a solution of dipeptide **18a** (52.0 mg, 0.07 mmol) in DMF (0.5 mL), imidazole (29.3 mg, 0.43 mmol) and *t*-butyldimethylsilyl chloride (32.4 mg, 0.21 mmol) were added and the resulting mixture was stirred at rt After 20 h, the solvent was eliminated under reduced pressure and the residue was taken up in ethyl acetate, washed with water and brine, dried filtered and evaporated in vacuo. The residue was purified by flash column chromatography (ethyl acetate/hexane 1:3) to afford dipeptide **18b** (56.1 g, 93%) as a clear oil. ^1^H-NMR (CDCl_3_, 250 MHz, ppm): 0.13, 0.18 (2 × s, 6H, -Si(CH_3_)_2_), 0.92 (s, 9H, -SiC(CH_3_)_3_), 1.38 (s, 9 H, -C(CH_3_)_3_), 2.94–3.22 (m, 3H), 3.57 (s, 3H, OCH_3_), 3.71–4.04 (m, 3H), 4.16–4.98 (m, 8H); 5.01 (bs, 1H, Pcp-NH), 6.50 (bs, 1H, Tyr-NH), 6.82–7.10 (m, 4H, 4 × HAr), 7.26–7.36 (m, 15H, 15 × HAr). ^13^C-NMR (CDCl_3_, 62.5 MHz, ppm): −5.3, −4.3, 17.9, 28.3, 37.7, 49.3, 53.0, 55.8, 69.8, 72.0, 72.3, 80.0, 80.3, 84.9, 87.3, 114.9, 127.0, 127.4, 127.6, 127.7, 127.9, 128.3, 128.4, 128.5, 130.4, 136.9, 137.6, 138.2, 156.0, 157.4, 171.0, 172.1. HRMS (ESI^+^) calculated for C_48_H_64_N_2_O_9_Si [M + 2H]^+^: 840.4370. Found: 840.4381.

#### 3.1.7. (*1S,2R,3S,4S,5R*)-5-[(*N*-tert-butoxycarbonyl-*O*-benzyloxycarbonyl)-l-tyrosylamino]-2,4-dibenzyloxy-3-tert-butyldimethylsilyloxy-1-(metoxy-*L*-prolyl-*L*-phenylalanylcarbonyl) Cyclopentane (**19**)

To a solution of dipeptide **18b** (56.1 mg, 0.07 mmol) in THF (0.65 mL), a solution of lithium hydroxide (14.1 mg) in methanol/water 2:1 (0.9 mL) was added and the resulting mixture was stirred at rt After 4 h, the reaction mixture was acidified with DOWEX 50W, filtered and the filtrate was evaporated under reduced pressure. To a solution of the resulting residue in DMF (0.2 mL), a solution of Phe-Pro-OMe (19.3 mg, 0.07 mmol) in DMF (0.2 mL), TBTU (25.3 mg 0.08 mmol) and Et_3_N (20 µL) were added and the mixture was stirred at rt After 20 h, the solvents were eliminated under reduced pressure and the residue was purified by flash column chromatography (dichloromethane/methanol 95:5), to obtain tetrapeptide **19** (33.8 mg, 47%) as a white solid. ^1^H-NMR (CDCl_3_, 250 MHz, ppm): 0.14, 0.18 (2 × s, 6H, -Si(CH_3_)_2_), 0.91 (s, 9H, -SiC(CH_3_)_3_), 1.34 (s, 9H, -C(CH_3_)_3_), 1.71–1.88 (m, 4H), 2.81–3.14 (m, 4H), 3.65 (s, 3H, OCH_3_), 3.69–3.82 (m, 3H), 4.09–4.22 (m, 4H), 4.25–4.43 (m, 5H), 4.63–4.95 (m, 4H); 5.10 (bs, 1H, NH), 6.85–7.10 (m, 5H, 4 × HAr, 2 × NH), 7.22–7.41 (m, 20H, 20 × HAr). ^13^C-NMR (CDCl_3_, 62.5 MHz, ppm): −4.7, −4.5, 18.1, 25.0, 28.2, 29.1, 37.2, 38.5, 40.8, 46.2, 51.1, 55.2, 52.4, 54.9, 58.9, 59.1; 70.08, 72.3, 72.5, 72.6, 80.2, 85.7, 86.9, 115.1, 125.2, 127.3, 127.7, 128.1, 128.3, 128.4, 128.6, 130.6, 128.4, 128.5, 130.4, 135.9, 136.4, 137.2, 138.2, 155.3, 157.9, 170.0, 170.7, 171.5, 172.4. HRMS (ESI+) calculated for C_62_H_79_N_4_O_11_Si [M + H]^+^: 1083.5509. Found: 1083.5493.

#### 3.1.8. (*1S,2R,3S,4S,5R*)-5-(*O*-benzyloxycarbonyl)-l-tyrosylamino-2,4-dibenzyloxy-1-(amido-l-prolyl-l-phenylalanylcarbonyl) Cyclopentane (**5b**)

To a solution of tetrapeptide **19** (32.5 mg, 0.03 mmol) in THF (0.3 mL), a solution of lithium hydroxide (6.4 mg, 0.4 mL) in methanol/water 2:1 was added and the mixture was stirred at rt. After 4 h, the reaction mixture was acidified with DOWEX 50W, filtered and the filtrate evaporated under reduced pressure. To a solution of the obtained acid 20 in THF (1 mL), ethyl chloroformate (3.10 mg, 0.03 mmol) and Et_3_N (6 µL) were added. After stirring the resulting mixture for 1 h, NH_3_ (30% in water, 0.6 µL, 0.03 mmol) was added and the resulting mixture was stirred overnight. The solvents were removed under reduced pressure and the residue was filtered through a pad of silica gel eluting with dichlorometane/methanol (95:5) to obtain a white solid, which was then dissolved into TFA (0.4 mL) and CH_2_Cl_2_ (0.6 mL). After stirring the resulting mixture for 4 h, the solvents were removed *in vacuum* and the residue was co-evaporated with toluene, to afford tetrapeptide **5b** (10.2 mg, 40%) as an amorphous white solid. ^1^H-NMR (CDCl_3_, 250 MHz, ppm): 1.72–1.88 (m, 4H), 2.74–3.13 (m, 4H), 3.71–4.22 (m, 7H), 4.21–4.48 (m, 5H), 4.63–4.95 (m, 4H); 6.85–7.10 (m, 5H, 4 × HAr, 2 × NH), 7.17–7.45 (m, 22H, 20 × HAr, 2 × NH). ^13^C-NMR (CDCl_3_, 62.5 MHz, ppm): 24.8, 28.9, 36.5, 38.0, 46.7, 51.8, 52.2, 52.4, 54.9, 58.9, 69.9, 71.6, 71.8, 80.2, 81.3, 85.6, 87.5, 114.9, 127.0, 127.4, 127.6, 127.7, 127.9, 128.3, 128.4, 128.5, 130.4, 135.6, 136.9, 137.8, 138.0, 169.8, 170.7, 172.1, 173.8. HRMS (ESI+) calculated for C_50_H_56_N_5_O_8_ [M + H]^+^: 854.4123. Found: 854.4133.

#### 3.1.9. (*1S,2R,3S,4S,5R*)-5-(l-tyrosylamino)-1-(amido-Prolyl-l-phenylalanyloxycarbonyl) Cyclopentane (**5a**)

To a solution of tetrapeptide **5b** (8.2 mg, 0.01 mmol) in methanol (1 mL), Pd(OH)_2_ (1 mg) was added and the mixture was stirred under hydrogen atmosphere at P = 20 atm. After 24 h, the reaction mixture was filtered through a pad of celite and the filtrate was evaporated under reduced pressure, obtaining tetrapeptide **5a** (5.5 mg, 98%) as a white solid. Data for the major conformer: ^1^H-NMR (DMSO-*d*_6_, 500 MHz, ppm): 1.73 (m, 1H, Pro-H4a), 1.76 (m, 1H, Pro-H4b), 1.79 (m, 1H, Pro-H3b), 1.93 (m, 1H, Pro-H3a), 2.45 (m, 1H, Tyr-CHAr), 2.73 (m, 1H, Phe-CHPh), 2.87 (m, 1H, Tyr-CHPh), 2.88 (m, 1H, Pcp-H2), 3.00 (m, 1H, Phe-CHPh), 3.14 (m, 1H, Pro-H5b), 3.46 (m, 1H, Pcp-H4), 3.57 (m, 1H, Pro-H5a), 3.68 (m, 1H, Pcp-H5), 3.75 (m, 1H, Tyr-H2), 3.84 (m, 1H, Pcp-H3), 4.15 (m, 1H, Pcp-H6), 4.20 (m, 1H, Pro-H2), 4.55 (m, 1H, Phe-H2), 5.10 (m, 1H, OH), 5.16 (m, 1H, OH), 5.19 (m, 1H, OH), 6.71 (m, 2H, 2 × HAr), 6.86–7.21 (m, 2H, CONH_2_), 7.04 (m, 2H, 2 × HAr), 7.12–7.17 (m, 5H, 5 × HAr), 8.20 (bs, 1H, Pcp-NH), 8.46 (m, 1H, Phe-NH), 9.45 (m, 1H, Tyr-OH). ^13^C-NMR (DMSO-d_6_, 62.5 MHz, ppm): δ 24.8, 29.4, 37.4, 37.5, 37.7, 41.9, 47.0, 53.0, 56.5, 60.1, 76.8, 78.7, 81.9, 115.8, 126.8, 128.6, 128.8, 130.8, 137.8, 156.9, 169.6, 170.1, 171.6, 173.7. HRMS (ESI+) calculated for C_29_H_38_N_5_O_8_ [M + H]^+^: 584.2715. Found: 584.2723.

### 3.2. Conformational Analysis

**NMR:** Peptidomimetic **5a** (3 mg) was dissolved in DMSO-*d*_6_ (0.55 mL) in a 5 mm NMR tube. ^1^H and ^13^C NMR assignments were determined from standard 1D and 2D COSY, TOCSY, ROESY, HSQC and HMBC experiments that were recorded on a Bruker Avance III HD 500 MHz spectrometer equipped with a QCI-P CryoProbe™ (proton-optimized quadruple resonance NMR ‘inverse’ probe). Proton-proton interatomic distances were estimated from the 2D ROESY spectrum recorded with a mixing time of 500 ms. The solution structure was calculated by restrained molecular dynamics with the program XPLOR-NIH (version 2.44.6, Center for Information Technology, National Institutes of Health, Bethesda, MD, USA) [31]. ROESY cross-peaks were classified as strong, medium or weak according to their intensities, and used as distance restraints in the structure calculation.

**Docking studies:** To perform the docking studies and analyze the binding modes of the tetrapeptide ligands to MOR, the Lamarckian genetic algorithm implemented in the Auto-dock 4.2 [32] program was used through PYMO [33]. The crystallized MOR complex obtained from the Protein Data Bank (PDB ID: 6DDF) [34] was used for the present study. All non-polar hydrogens were removed from each ligand and Gasteiger partial charges [35] were assigned for all ligands. Polar receptor hydrogens were added and Kollman charges [36], along with atomic solvation parameters, were assigned to the individual protein atoms with AutoDock-Tools. For the docking, 45 × 45 × 45 Å3 grids with points separated by 0.375 Å were generated using the AutoGrid program. The grid was centered around the crystal structure of the corresponding agonist (Damgo). In total, 100 docking runs were performed and 50 solutions were clustered in groups with RMSD < 1 Å. These clusters were subsequently ranked by the lowest energy representative of each group. To analyze and visualize the resulting ligand–receptor complexes, the academic version of the Maestro Schrödinger suite v.12.3 program (Schrödinger, LLC, New York, NY, USA) was used [37].

## 4. Conclusions

We have developed an optimized synthesis of a polyhydroxylated 2-aminocyclopentanecarboxylic acid using a key step consisting of an intramolecular C-alkylation of an in situ generated nitronate. The resulting amino acid could be a useful scaffold for the construction of bioactive peptides. To prove this hypothesis, we have prepared the morphiceptin peptidomimetic **5a**, incorporating this new cyclopentane β-amino acid in place of the central proline residue. In addition, tetrapeptide **5a** was modeled by using in silico methods to reveal the mechanism of the interaction with the MOR receptor. These studies suggest a more effective binding of novel peptidomimetic **5a** to the receptor than the parent morphiceptin. Accordingly, this tetrapeptide might have an important potential for the development of clinically useful drugs for pain treatment. Studies aimed towards fully delineating the physical chemical and biological properties of this peptidomimetic potential drug candidate are currently under investigation in our laboratory.

## Supplementary Materials

The following are available online, NMR spectra of compounds **3b**, **5a**, **9**, **12** and **18a**.

## Abbreviations

TBAF	Tetrabuthyl Ammonium Fluoride
THF	Tetrahydrofuran
TFA	Trifluoroacetic Acid
TBTU	2-(1H-Benzotriazole-1-yl)-1,1,3,3-tetramethylaminium tetrafluoroborate
ESI-HRMS	Electrospray Ionization High Resolution Mass Spectrometry
DIEA	*N*,*N*-Diisopropylethylamine
DMF	for *N*,*N*-Dimethylformamide
NOE	Nucelar Overhouse Effect
DOWEX	*DOWEX** ion exchange resins
COSY	COrrelation SpectroscopY
TOCSY	TOtal Correlation SpectroscopY
ROESY	Rotating-frame Overhauser Effect SpectroscopY
HSQC	Heteronuclear Simple Quantum Coherence
HMBC	Heteronuclear Multiple Bond Correlation
RMSD	Root-Mean-Square Deviation
