# Supplementary material for: New Morphiceptin Peptidomimetic Incorporating (1S,2R,3S,4S,5R)-2-Amino-3,4,5-trihydroxycyclopen-tane-1-carboxylic acid: Synthesis and Structural Study
Source: Molecules. 2020 Jun 1;25(11):2574. doi: 10.3390/molecules25112574 (PMC7321350; doi:10.3390/molecules25112574)
Supplement: Supplementary file 1 [file molecules-25-02574-s001.pdf]

Supporting Information

New morphiceptin peptidomimetic incorporating (1*S*,2*R*,3*S*,4*S*,5*R*)-2-amino-3,4,5-trihydroxycyclopentane-1-carboxylic acid: synthesis and structural study

Raquel Soengas[1], Marcos Lorca[1,3], Begoña Pampín[1], Víctor M. Sánchez-Pedregal[2], Ramón J, Estévez[1], Juan C. Estévez[1,*]

[1] *Centro Singular de Investigación en Química Biolóxica e Materiais Moleculares (CIQUS), Departamento de Química Orgánica, Campus Vida, Universidade de Santiago de Compostela, 15782, Santiago de Compostela*
[2] *Departamento de Química Orgánica, Campus Vida, Universidade de Santiago de Compostela, 15782, Santiago de Compostela*
[3] *Instituto de Química y Bioquímica, Facultad de Ciencias, Universidad de Valparaíso, Av. Gran Bretaña 1111, Valparaíso 2360102, Chile.*

Contents

NMR spectra of the synthetic intermediates S2
Figure S1. NMR spectra of **9**.
Figure S2. NMR spectra of **12**.
Figure S3. NMR spectra of **3b**.
Figure S4. NMR spectra of **18a**.
NMR spectra of the morphiceptin peptidomimetic S6
Figure S5. ^{1}H NMR spectrum of **5a**.
Figure S6. ^{13}C NMR spectrum of **5a**.
Figure S7. ^{1}H,^{1}H COSY NMR spectrum of **5a**.
Figure S8. ^{1}H,^{13}C HSQC NMR spectrum of **5a**.
Figure S9. ^{1}H,^{13}C HMBC NMR spectrum of **5a**.
Figure S10. ^{1}H,^{1}H TOCSY NMR spectrum of **5a**.
Figure S11. ^{1}H,^{1}H ROESY NMR spectrum of **5a**.
Table S1. NMR assignment of peptidomimetic **5a**.
Table S2. Distance restraints of peptidomimetic **5a** derived from the 2D ROESY spectrum.
Figure S12. Summary of NOE contacts detected in the ROESY spectrum of compound **5a**.

(5*R*,6*S*,7*R*)-6,7-Dibenzyloxy-5-*t*-butoxycarbonylamine-2-oxabicyclo-[2.2.1]heptan-3-one (9)

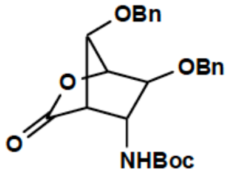

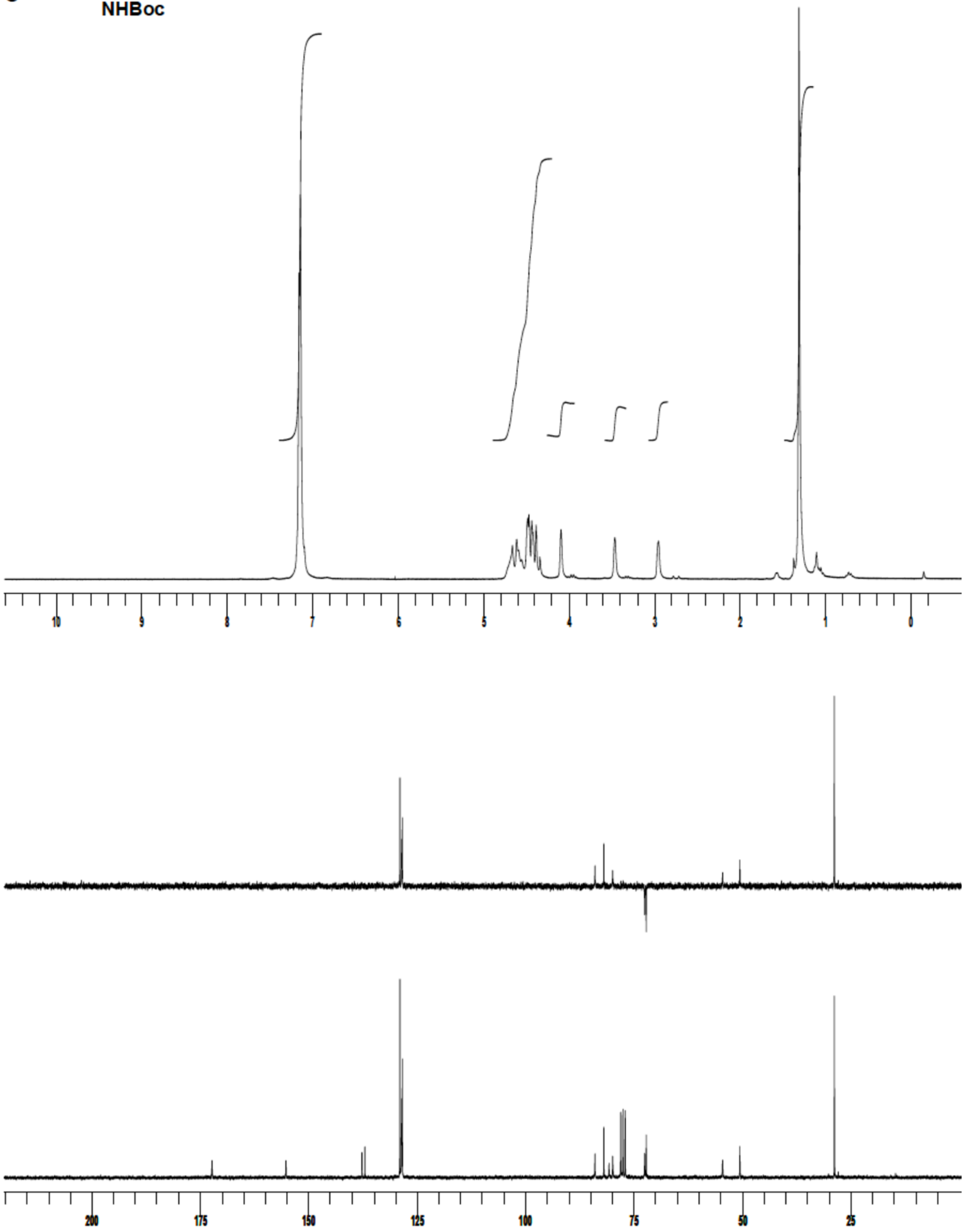

Figure S1. NMR spectra of **9**.

(3*S*,5*R*,6*S*,7*R*)-6,7-Dibenzyloxy-3-methoxy-5-nitro-2-oxabicycle[2.2.1]heptane (12)

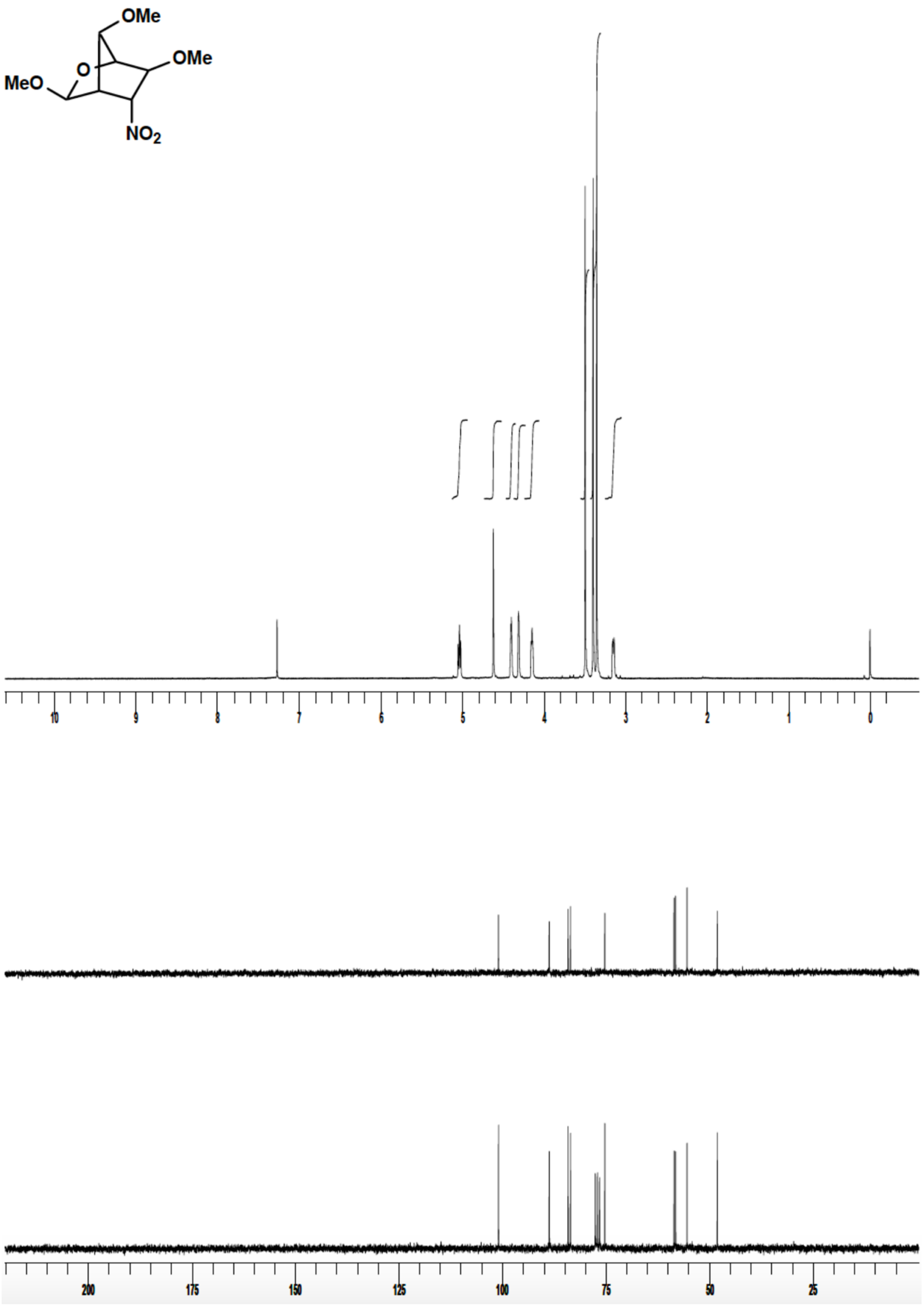

Figure S2. NMR spectra of **12**.

Methyl (1*S*,2*R*,3*S*,4*S*,5*R*)-2,4-dibenzyloxy-5-*t*-butoxycarbonylamine-3-hydroxycyclopentanecarboxylate (pcp) (3b)

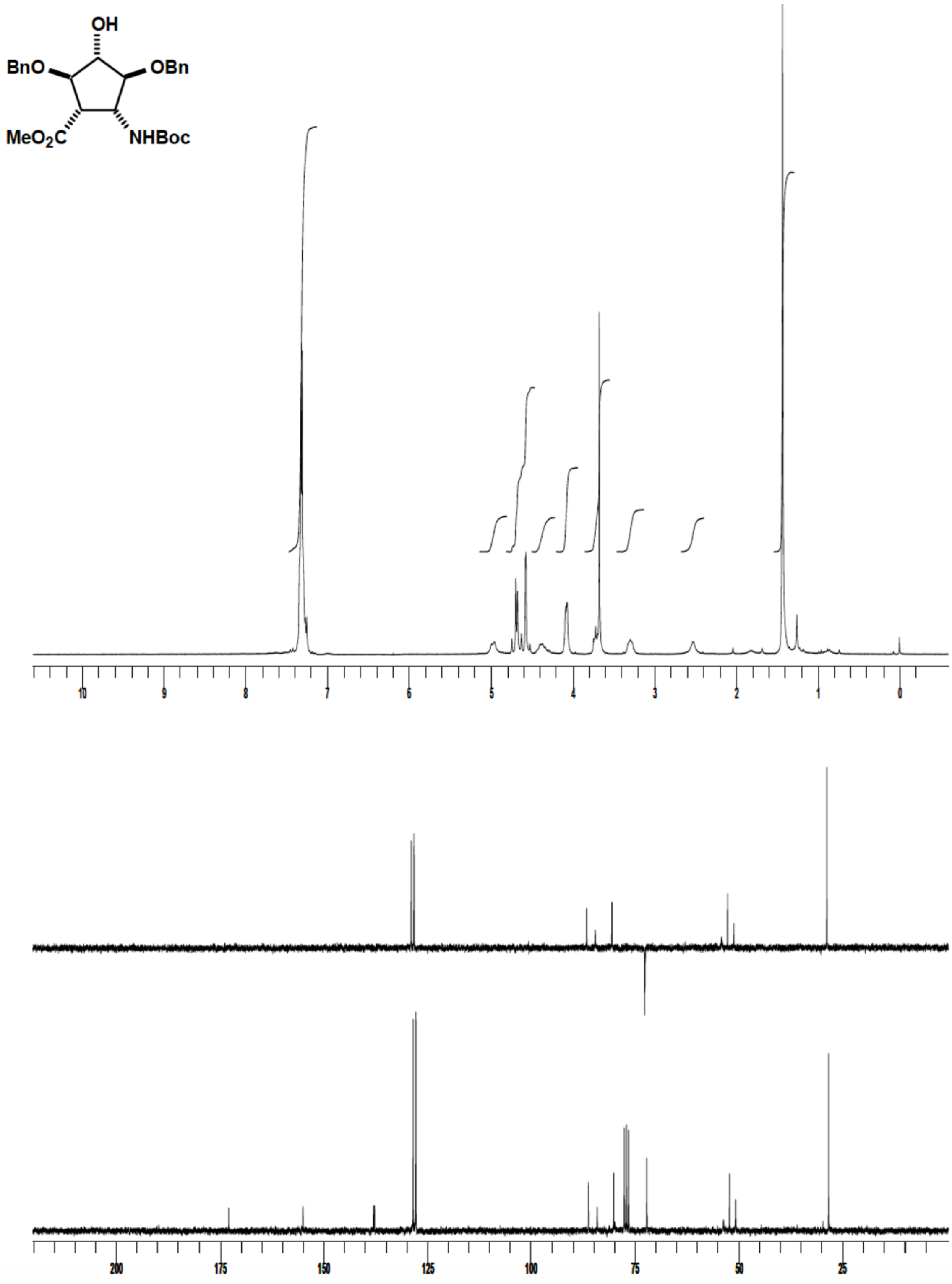

Figure S3. NMR spectra of **3b**.

Methyl (1*S*,2*R*,3*S*,4*S*,5*R*)-5-(*N*-*tert*-butoxycarbonyl-*O*-benzyloxycarbonyl-L-tyrosylamino)-2,4-dibenzyloxy-3-hydroxy-cyclopentanoate (18a)

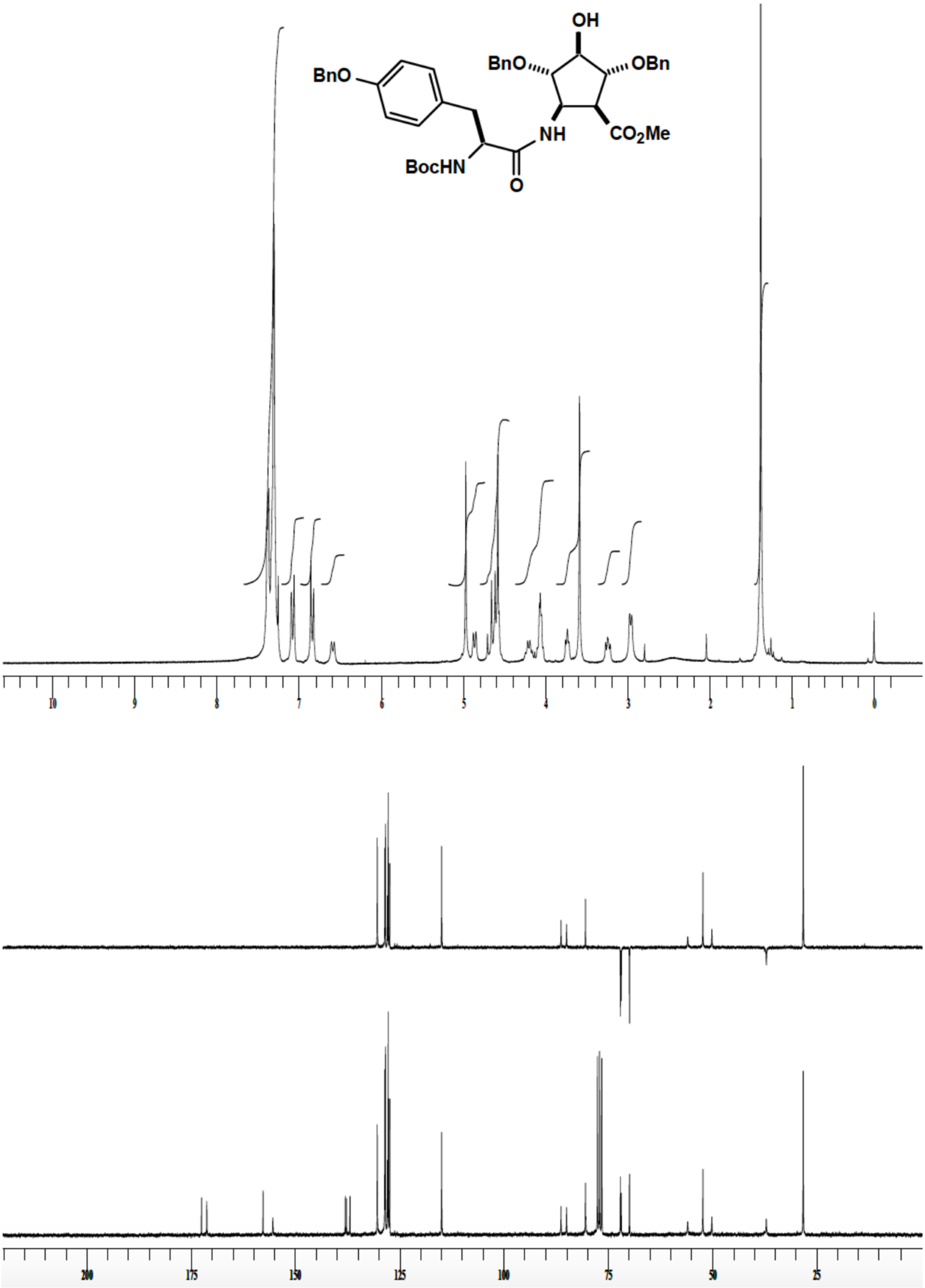

Figure S4. NMR spectra of **18a**.

NMR spectra of the morphiceptin peptidomimetic (DMSO-d6, 500 MHz)

(1*S*,2*R*,3*S*,4*S*,5*R*)-5-(L-Tyrosylamino)-1-(amido-prolyl-L-phenylalanyloxycarbonyl) cyclopentane (5a)

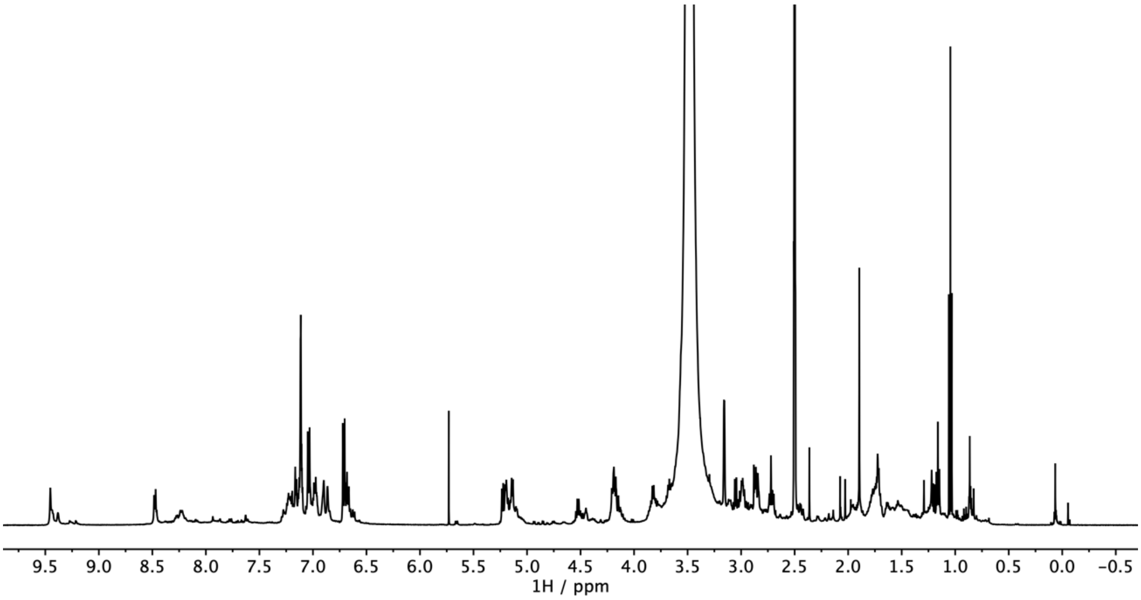

Figure S5. ^{1}H NMR spectrum of **5a** (DMSO-d6, 500 MHz, 298 K).

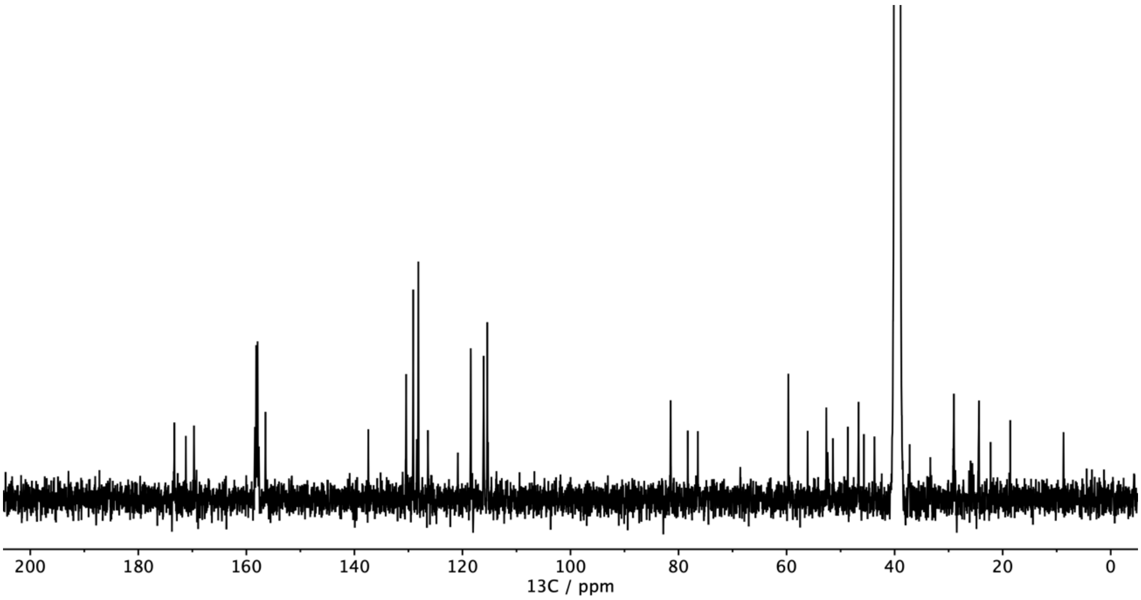

Figure S6. ^{13}C NMR spectrum of **5a** (DMSO-d6, 500 MHz, 298 K).

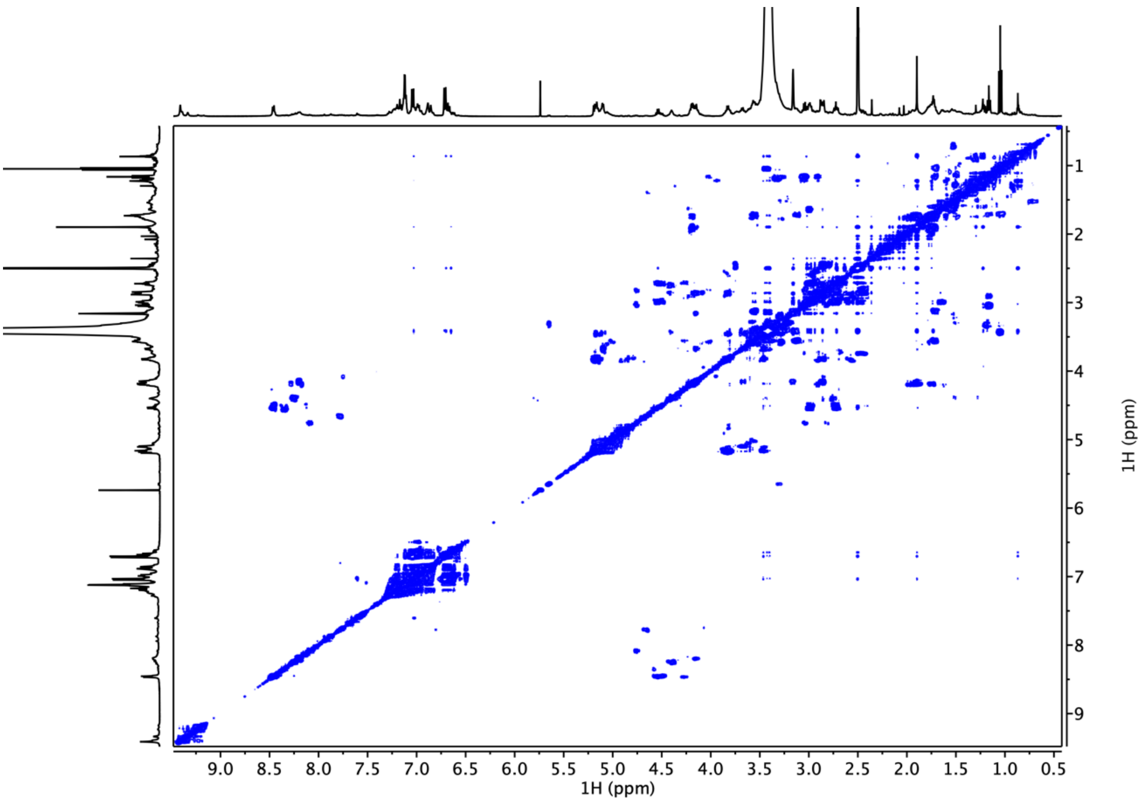

Figure S7. COSY NMR spectrum of **5a** (DMSO-d6, 500 MHz, 298 K).

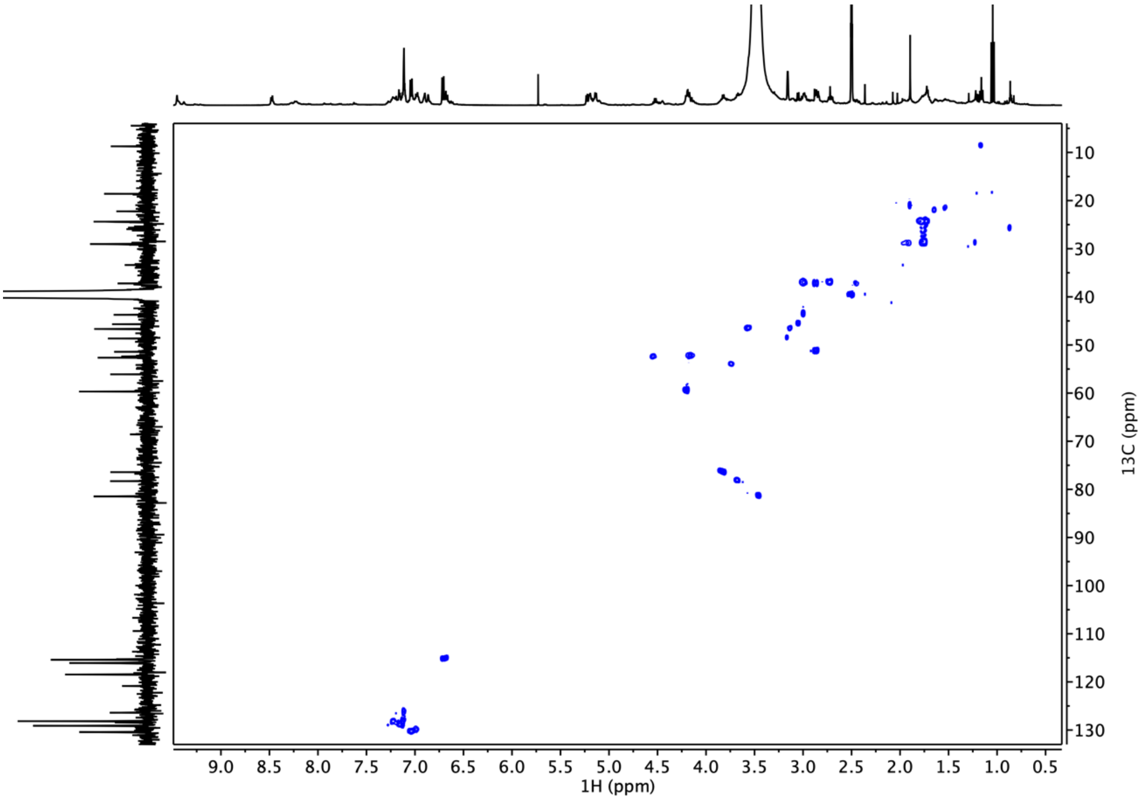

Figure S8. ^{13}C-^{1}H HSQC NMR spectrum of **5a** (DMSO-d6, 500 MHz, 298 K).

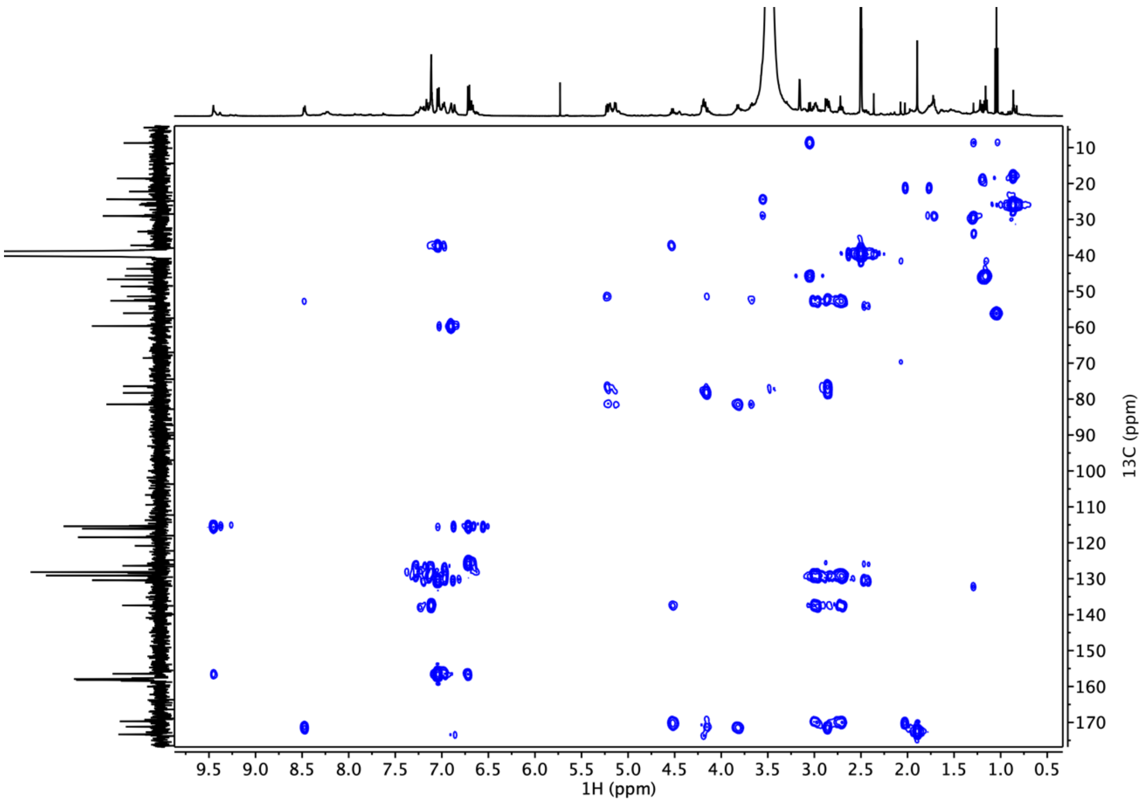

Figure S9. HMBC NMR spectrum of **5a** (DMSO-d6, 500 MHz, 298 K).

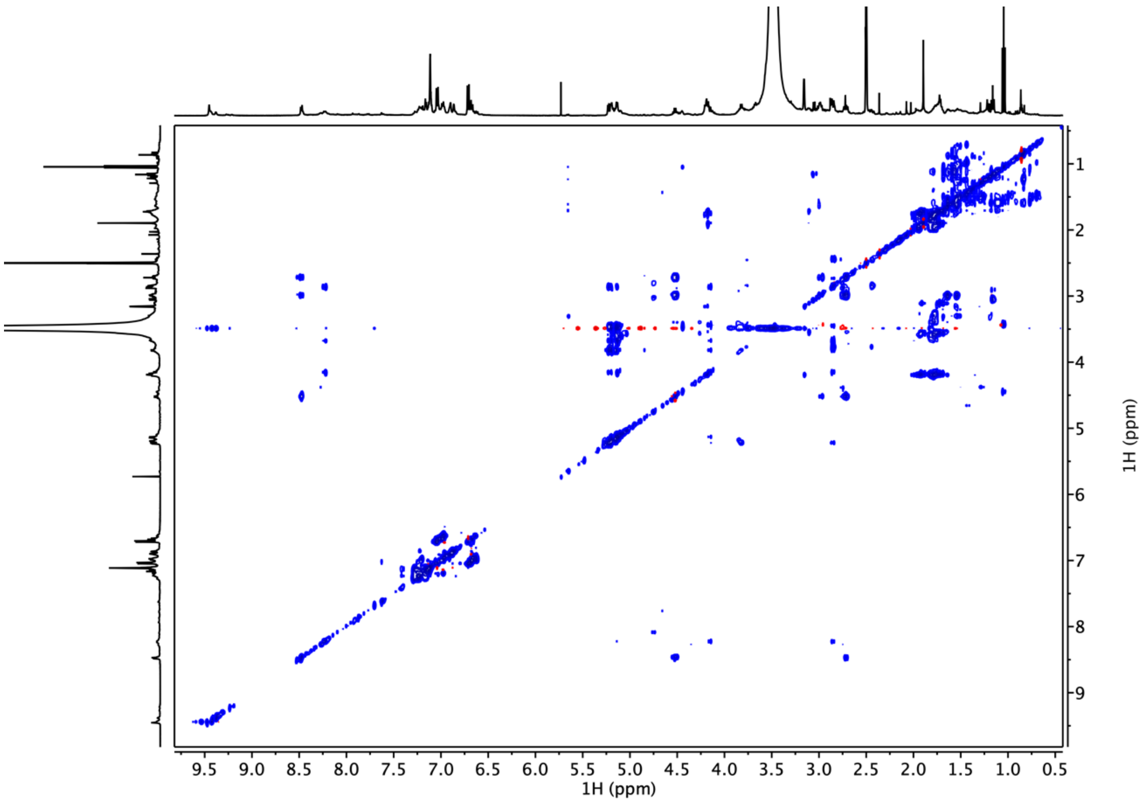

Figure S10. TOCSY NMR spectrum of **5a**, mixing time 50 ms (DMSO-d6, 500 MHz, 298 K).

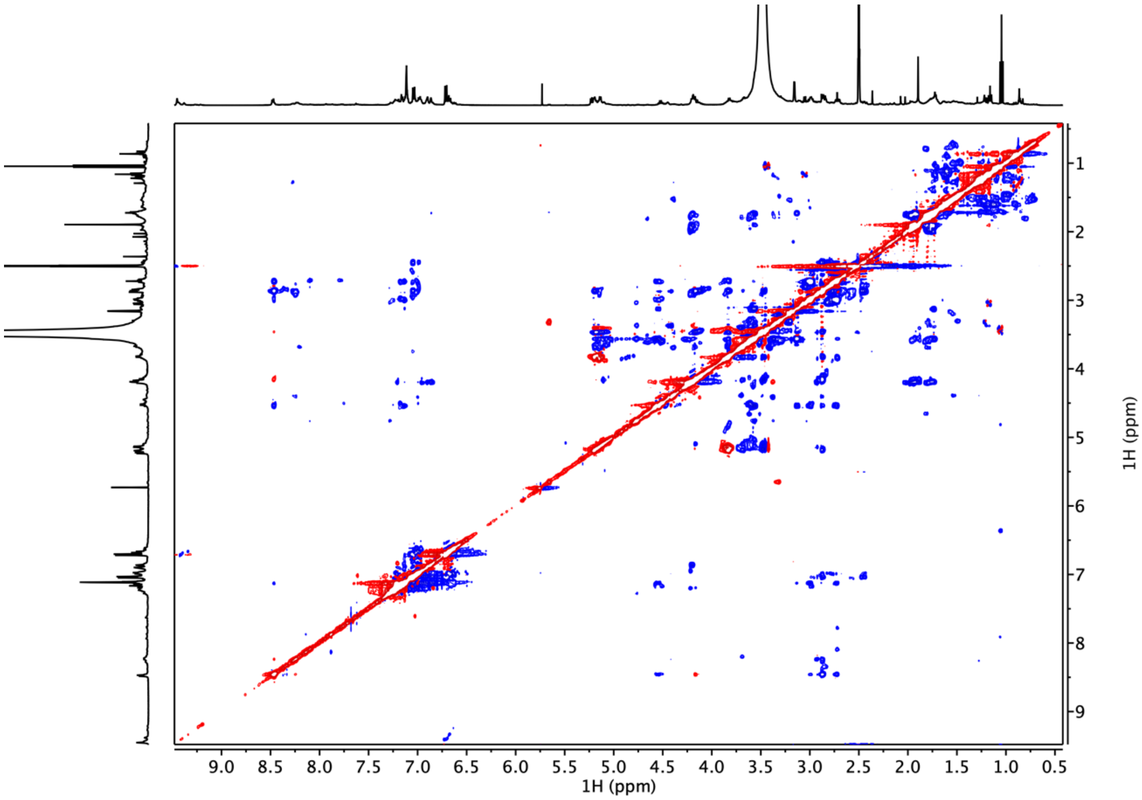

Figure S11. ROESY NMR spectrum of **5a**, mixing time 500 ms (DMSO-d6, 500 MHz, 298 K).

Table S1. NMR assignment of peptidomimetic **5a** (DMSO-d6, 500 MHz, 298 K).

residue	C atom	δ_C / ppm	H atom	δ_H / ppm
Tyr 1	N	-	HN	-
	C(O)	169.23	-	-
	CA	54.12	HA	3.75
	CB	37.45	HB1, HB2	2.45, 2.87
	CG	125.99	-	-
	CD	130.42	HD1, HD2	7.04
	CE	115.40	HE1, HE2	6.71
	CZ	156.46	-	-
	OH	-	HH	9.45
Pcp 2	N	-	HN	8.20
	C(O)	171.20	-	-
	CA	51.42	HA	2.88
	CB	52.39	HB	4.15
	CG	78.29	HG2	3.68
	CD	81.46	HD1	3.46
	CE	76.42	HE2	3.84
	OG	-	HOG	5.10
	OD	-	HOD	5.16
	OE	-	HOE	5.19
Phe 3	N	-	HN	8.46
	C(O)	169.70	-	-
	CA	52.64	HA	4.55
	CB	37.22	HB1, HB2	2.73, 3.00
	CG	137.43	-	-
	CD	129.11	HD1, HD2	7.12-7.17
	CE	128.16	HE1, HE2	7.12-7.17
	CZ	126.40	HZ	7.12-7.17
Pro 4	N	-	-	-
	C(O)	173.33	-	-
	CA	59.67	HA	4.20
	CB	29.03	HB1	1.93
			HB2	1.76
	CG	24.38	HG2	1.79
			HG1	1.73
	CD	46.67	HD1	3.57
			HD2	3.14
C-ter	N		HN1, HN2	6.86-7.21

Table S2. Distance restraints of peptidomimetic **5a** derived from the 2D ROESY spectrum (t-mix 500 ms, DMSO-d6, 500 MHz, 298 K). Cross-peaks intensities were classified as *S* strong, *M* medium, *W* weak. Distance boundaries were set to 1.8-2.5 Å (S), 2.5-3.5 Å (M) and 3.5-5.0 Å (W).[1]

	atom 1	atom 2	class
Inter-residue	PCP:HA	PHE:HN	M
	PCP:HE2	PHE:HB2	M
	PHE:HA	PRO:HD1	S
	PHE:HA	PRO:HD2	M
Tyr 1	TYR:HD1 or HD2	TYR:HB1	M
	TYR:HD1 or HD2	TYR:HB2	M
Pcp 2	PCP:HA	PCP:HB	S
	PCP:HA	PCP:HD1	M
	PCP:HB	PCP:HD1	M
	PCP:HB	PCP:HG2	M
	PCP:HE2	PCP:HA	M
	PCP:HE2	PCP:HG2	M
	PCP:HE2	PCP:HD1	M
Phe 3	PHE:HN	PHE:HA	M
	PHE:HN	PHE:HB1	W
	PHE:HN	PHE:HB2	M
	PHE:HA	PHE:HB1	M
	PHE:HA	PHE:HB2	M
	PHE:HD1 or HD2	PHE:HA	M
	PHE:HD1 or HD2	PHE:HB1	M
	PHE:HD1 or HD2	PHE:HB2	M
Pro 4	PRO:HA	PRO:HB1	S
	PRO:HA	PRO:HB2	M
	PRO:HD1	PRO:HG1	S
	PRO:HD1	PRO:HB1	M
	PRO:HD2	PRO:HG1	M

[1] Markley J L, Bax A, Arata Y, Hilbers C W, Kaptein R, Sykes B D, Wright P E, Wüthrich. K. Recommendations for the presentation of NMR structures of proteins and nucleic acids. J Mol Biol. 1998;280: 933–952. DOI:10.1006/jmbi.1998.1852.

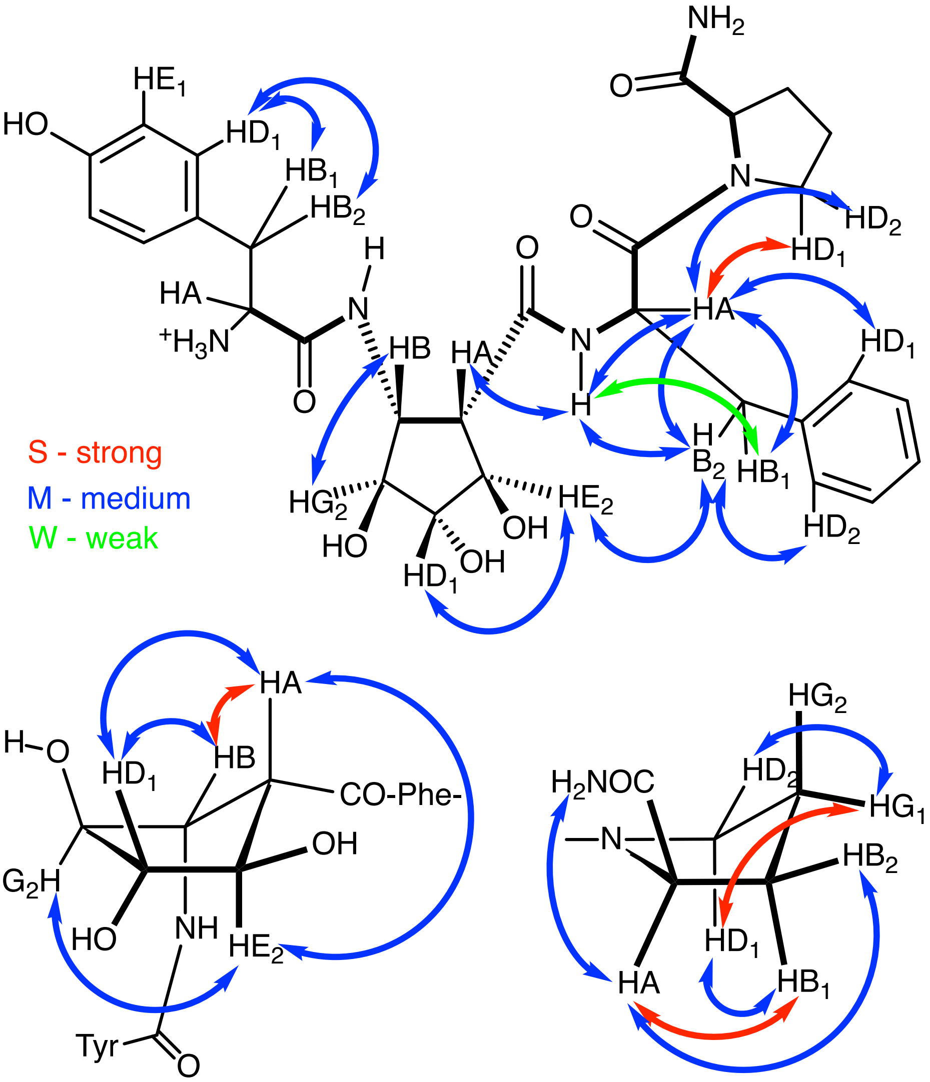

Figure S11. Summary of NOE contacts detected in the ROESY spectrum of compound **5a**. Distance class is encoded with colours.
